# Mite bombs or robber lures? The roles of drifting and robbing in *Varroa destructor* transmission from collapsing honey bee colonies to their neighbors

**DOI:** 10.1371/journal.pone.0218392

**Published:** 2019-06-21

**Authors:** David Thomas Peck, Thomas Dyer Seeley

**Affiliations:** Department of Neurobiology and Behavior, Cornell University, Ithaca, New York, United States of America; University of California San Diego, UNITED STATES

## Abstract

When honey bee colonies collapse from high infestations of Varroa mites, neighboring colonies often experience surges in their mite populations. Collapsing colonies, often called “mite bombs”, seem to pass their mites to neighboring colonies. This can happen by mite-infested workers from the collapsing colonies drifting into the neighboring colonies, or by mite-free workers from the neighboring colonies robbing out the collapsing colonies, or both. To study inter-colony mite transmission, we positioned six nearly mite-free colonies of black-colored bees around a cluster of three mite-laden colonies of yellow-colored bees. We then monitored the movement of bees between the black-bee and yellow-bee colonies before, during, and after mite-induced collapse of the yellow-bee colonies. Throughout the experiment, we monitored each colony's mite level. We found that large numbers of mites spread to the black-bee colonies (in both nearby and distant hives) when the yellow-bee colonies collapsed from high mite infestations and became targets of robbing by the black-bee colonies. We conclude that “robber lures” is a better term than “mite bombs” for describing colonies that are succumbing to high mite loads and are exuding mites to neighboring colonies.

## Introduction

The parasitic mite *Varroa destructor* is a recently speciated parasite of the western honey bee *Apis mellifera* [1]. This parasite feeds upon both juvenile and adult honey bees, and is known to transmit harmful viruses between bees [2,3]. Varroa mites are wingless, eyeless, and unable to crawl between widely spaced honey bee nests. Despite these limitations, honey bee colonies are almost universally infested with these mites, including managed colonies that have been recently purged of mites by use of chemical treatments [4, 5, 6, 7], and wild colonies spaced widely in forests [8].

Bees can ferry mites between colonies either indirectly or directly. Indirectly, a mite can move from one bee to a neutral location like a flower, and from there to a bee from another colony. Mites are certainly agile enough to achieve this [9] but this indirect mechanism is unlikely to move large numbers of mites between colonies. Instead, it is likely that most mite transmission occurs directly, when a bee flies between its nest and another colony’s nest while carrying a mite. Drifting, when a bee leaves its natal nest and takes up residence in another colony’s nest, presents such an opportunity. Robbing, when a bee enters another colony's nest to steal honey and then carries it back to her own nest, offers another route of bee-mediated mite transmission. Worker bees can drift and rob, while drone bees can only drift. Drifting allows unidirectional mite transmission from the drifting bee’s original colony to its new colony. Robbing allows for bidirectional transmission; mites in a robbing colony can ride the robbers into a robbed colony’s nest, and mites in a robbed colony can climb onto the robbers and ride them to their home.

Our study focused on understanding the mechanisms underlying the widely reported phenomenon that when a colony dies suddenly ("collapses") with a large population of mites, the mite populations in neighboring colonies often skyrocket at roughly the same time [10, 11]. This has been described as a “mite bomb” phenomenon whereby mites are propelled as “shrapnel” into neighboring colonies via the drift of infested workers and drones out of the dying colony [11]. It has been suggested that such drift may even be a manipulation of bee behavior by the mites themselves [12]. Another possible explanation for surging mite populations in colonies around collapsing colonies is that these colonies are "robber lures," and that it is healthy robber bees, not sick drifter bees, that disperse the mites.

Drifting between wild colonies is considered uncommon given the wide spacing of their nests [13, 14]. Meanwhile, drifting between apiary colonies is certainly common. Jay [15,16] found that when hives painted white were placed in rows spaced 1 m apart, between 4% and 96% of marked workers drifted into other colonies, depending upon wind direction, distance from landmarks, and the number of colonies in the row. Goodwin et al. [17] found only 0–3% worker drift between nearby colonies, while Pfeiffer and Crailsheim [18] found up to 90% worker drift to neighboring colonies, and they estimated that up to 40% of the workers in some apiary hives may have drifted in from elsewhere. Thus, worker drift in apiaries is quite common, though the amount of drift is highly variable. Drone drift of at least 50% has been measured at intercolony spacings typical of apiaries (<1m), but was barely detectable (0–2%) when colonies were separated by 40-100m [19]. Thus, drift of both workers and drones is common within apiaries, but is greatly reduced when colonies are spaced more widely.

Robbing between colonies has also been implicated as a mechanism of mite transmission. Sakofski et al. [5] reported highest mite invasion into treated colonies during the late summer, when robbing was most common in their study region. Greatti et al. [4] reported high rates of mite invasion during periods of nectar dearth, and hypothesized that robbing of feral colonies was the likely cause. Frey et al. [7] monitored mite invasion of mite-free colonies at various distances (1m to 1.5km) from mite-infested colonies and found no protective effect of distance, proposing that all colonies had robbed the mite-infested colonies during a nectar dearth. All of these studies measured the fall of dead mites below colonies that were continuously treated with miticides. One of us (TDS) [8] had previously presumed robbing to be unlikely among colonies living in the wild due to the wide spacing of forest colonies, but we have recently recorded bees from widely spaced forest colonies quickly discovering and robbing from unguarded honey combs in their environment. Thus, robbing may be common even across large distances.

Our goal was to determine whether drifting, robbing, or a combination of the two best explains mite transmission from dying, mite-infested colonies to healthy, nearly mite-free colonies. If drifting is the primary mechanism, then we predicted *gradual* increases in the phoretic mite levels in the healthy colonies nearby as infested workers and drones drift into these colonies at more or less constant rates. If robbing is the primary mechanism of mite transmission from dying colonies, then we predicted *sudden* increases in the phoretic mite levels in the healthy colonies coinciding with an onset of robbing.

Besides investigating the mechanisms of inter-colony mite transmission, we investigated the effect of inter-colony distance on mite transmission from dying to healthy colonies. We did this to see if a large distance confers protection to the healthy colonies by reducing the dispersal of mites from the dying colonies, whether by reducing drifting or lessening robbing, or both.

## Methods

### Study site

**Fig 1:**
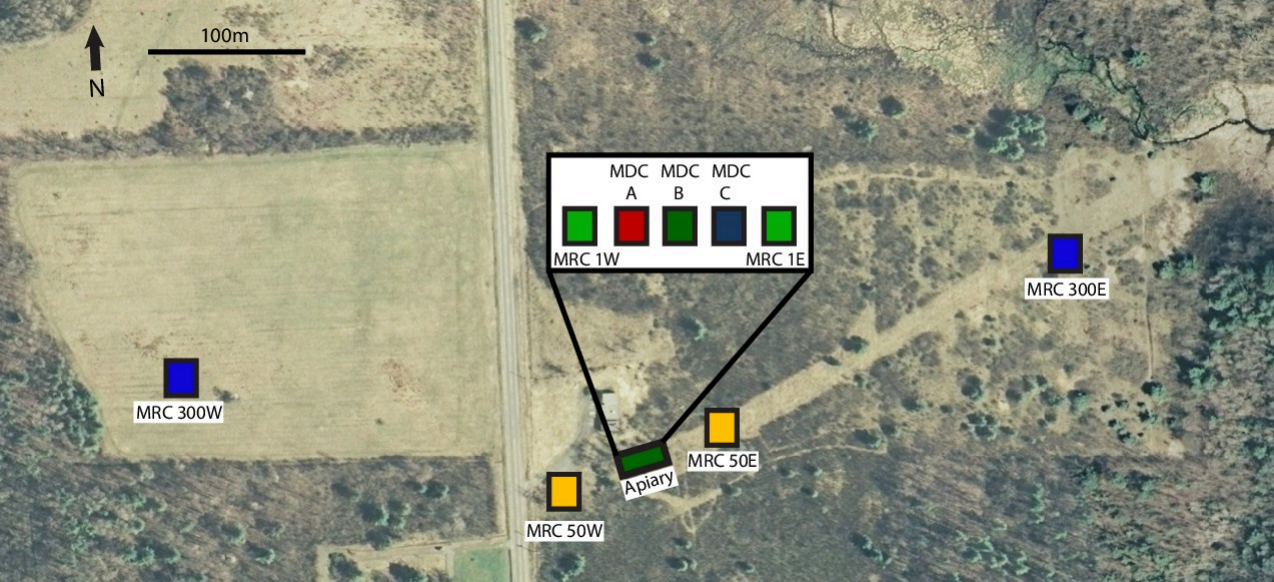
Birds-eye view of study site. It shows the array of three mite donor colonies (MDCs) and six mite receiver colonies (MRCs). All hive entrances faced south.

We conducted this experiment in a field near Ithaca NY (42°29'43.5"N 76°25'53.7"W). This location provided isolation from beekeepers' colonies and from wild colonies (the surrounding land cover is wetland and young forest). This site was used in a previous study of colony spacing and bee drifting [19]. As shown in Fig 1, we established an apiary containing three mite-donor colonies (MDCs) arranged in a line and spaced 0.5 m apart, and two mite-receiver colonies (MRCs) spaced 1m from the MDCs. Two more MRCs were placed 50m from the MDCs, in opposite directions, and another two MRCs were placed 300m from the MDCs, also in opposite directions. We used this symmetrical array to replicate our test of distance effects. We used three MDCs instead of one to ensure that at least one collapsed during the experiment, and to increase the total number of mites available to spread to the MRCs.

### Study colonies

To distinguish between resident and foreign bees at each hive, we used colonies with workers of two distinct colors: yellow and black. In mid-May 2017, we obtained 10 Cordovan Italian and 17 New World Carniolan queens from C.F. Koehnen and Sons, Inc. in California. The Cordovan queens (producing bright yellow offspring) were installed in nucleus colonies made from colonies with 1–3 mites per 300 bees (as determined by sugar shake [20]), which is a high level for mid-May. The New World Carniolan queens (producing dark brown or black offspring) were installed in nucleus colonies made from colonies with low mite counts (0–1 mites per 300 bees).

In the first week of June, the MDCs were moved into hives consisting of two full-depth, 10-frame hive bodies for a brood chamber, and one full-depth honey super (over a queen excluder). The MRCs were housed similarly, but were also given a screened bottom board for sampling the mites that would fall onto oiled boards. The hives were painted different colors to make them visually distinctive. To boost the mite populations in the MDCs, we provided each one with 4 frames of drone comb, so these colonies could rear numerous drones, the preferred hosts of the mites. Meanwhile, to minimize the mite populations in the MRCs, we provided each one with frames without drone comb. To preclude mite movement between MDCs and MRCs before the experiment began, we kept the two kinds of colonies in two apiaries located 3.5 km apart until we brought all the colonies together at the study site.

In early August, the three Cordovan colonies with the highest mite levels were chosen for the MDCs, and the six New World Carniolan colonies with the darkest workers were chosen for the MRCs. We considered continuously treating the MRCs with miticides so that any mites found in these colonies could be counted as immigrants from the MDCs (as per [4, 5, 7]). However, we did not do so, because continuous treatment could have left chemical residues repellent to mites, altered the behavior of the bees in the treated colonies, or tagged the MRC bees with an odor identifiable by guard bees in the MDCs. Withholding miticides from the MRCs also enabled us to monitor the MRCs for mite-induced colony mortality.

### Monitoring collapsing colonies

On 16 August 2017, we moved the three MDCs to the study site. The next morning, we moved the six MRCs to this site. Once all the colonies were installed at the study site, we placed an inverted hive cover on the ground in front of each hive in the apiary (Fig 1) to collect the objects the bees dragged from each hive. From this point on, we collected data at regular intervals through the end of November 2017. The specific dates of data collection had to be adjusted for weather, but our target schedule was as follows: (1) every 10 days, perform a sugar-shake count [20] of the phoretic mites in each colony (based on a volumetric sample of 300 dry workers from frames containing both capped and uncapped brood); (2) every 5 days, count the mites that had fallen onto oiled boards beneath the MRCs, distinguishing between adult (dark) and juvenile (light) mites; and (3) every 4 days (as weather permitted), count 100 workers and 100 drones entering or leaving each colony's hive, noting how many were of the "wrong" color for the colony. If fewer than 100 workers or drones were observed in 5 minutes, we noted the percent of off-color bees (e.g., yellow bees entering or leaving a dark-bee hive.) Besides collecting the data described above, we also inventoried the contents of the inverted hive cover sitting on the ground in front of each hive, to count the dead adult bees, immature bees, and mites ejected from each colony. These were counted exactly, except on days when we found more than 100 dead bees; then they were counted by tallying the number of groups of 5 individuals of each type (adult drone, pupal drone, etc.).

We also assessed whether or not robbing was occurring at each colony each time we performed the data collections just described. Specifically, we observed the entrance of each colony’s hive for two minutes and noted whether there was (1) no fighting between workers; (2) more than one but fewer than ten instances of fighting, but no other obvious sign of robbing; or (3) “intense robbing”. We noted “intense robbing” when we saw more than ten instances of worker-worker fighting within one minute and one or more of the following diagnostic signs of robbing: hairless bees (ones that had been fighting with guards) attempting to enter the hive; arriving bees performing distinctive, side-to-side flight maneuvers at the hive entrance [21, 22, 23]); and many (>20) dead, foreign-color bees lying in the inverted hive cover in front of a hive. The look of “intense robbing” is shown in the supplementary media file S1 Video. To reduce the risk of stimulating robbing when we opened the hives to perform sugar-shake mite assays, we worked quickly and avoided placing frames containing honey in front of hives.

During one bout of intense robbing of the MDCs, on 27 September, we tracked robber bees back to their home hives by slightly modifying a method devised in the 16^th^ century [24]. We dusted with powdered sugar all the workers (robbers and guards) at the entrance of each MDC, then we went to the entrance of each MRC and counted how many of 100 workers returning to it were dusted with powdered sugar. We conducted these counts twice, sugar-dusting workers at all three MDCs each time, just before we watched at each of the six MRCs for sugar-dusted workers coming home.

After late November, we halted most of our data collection, but on 20 December, 20 February, and 20 April we assessed colony survival by knocking on each MRC's hive and listening for bees buzzing.

Using the data collected, we described the patterns of mite counts and bee behavior. We conducted multi-way repeated measures ANOVAs to analyze the changes over time and with distance, and the interactions between the two, in both the levels of phoretic mites in each MRC and the counts of adult mites dropping from each MRC. In both cases, we considered distance as a categorical variable. Statistical values are reported with Greenhouse-Geisser correction, since our small replicate numbers preclude an attempt to confirm that our data meet the ANOVA’s assumption of sphericity. Then, acknowledging that the data from our small sample may not meet the assumptions for parametric tests, we followed our initial analysis with non-parametric tests. We performed Friedman tests to analyze changes over time in phoretic mite levels and adult mite drop counts, and Kruskal-Wallis tests to analyze effects of distance. We also analyzed the phoretic mite level data and the adult mite drop count data for a few key values: the peak phoretic mite level and the peak mite drop count recorded in each MRC, and the final phoretic mite levels recorded in each MRC. We performed one-way ANOVAs with distance from the MDCs as the categorical grouping variable. All statistical analyses were conducted using IBM SPSS Statistics for Windows (Version 24.0).

## Results

### Mite count dynamics

**Fig 2:**
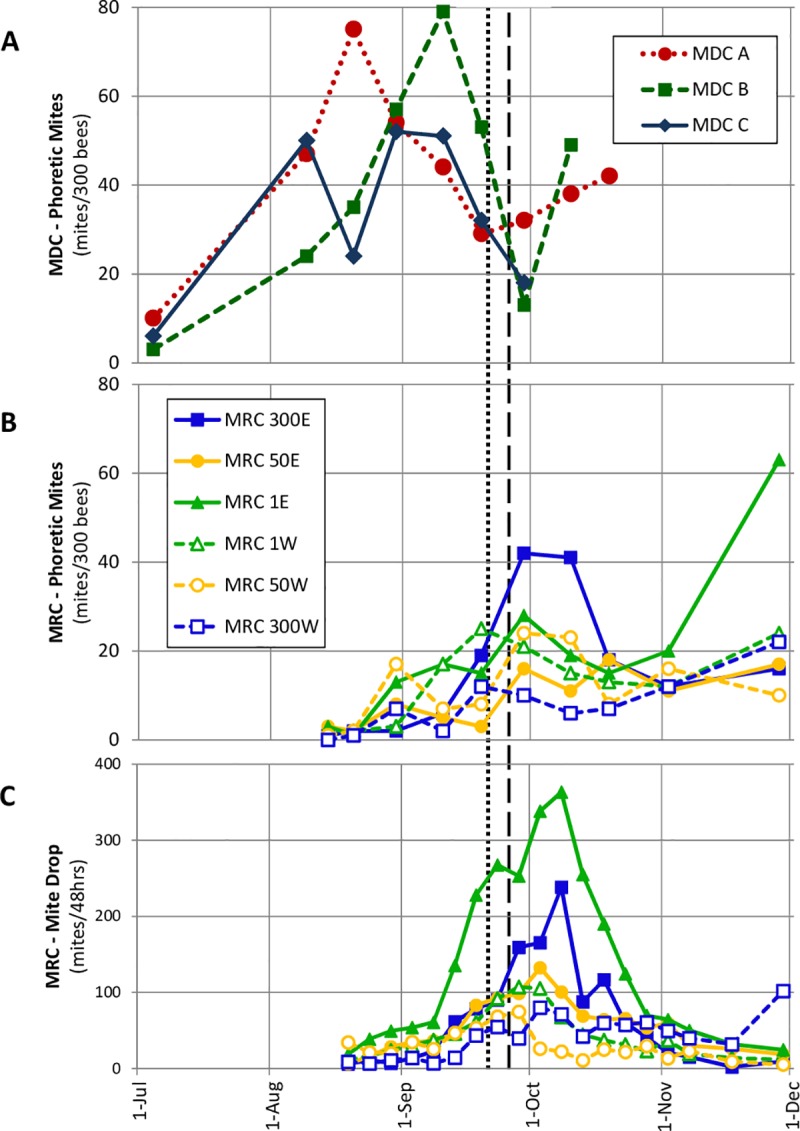
Measurements of mite levels in study colonies. Phoretic mite infestation levels for MDCs (A) and MRCs (B), and rates of adult (dark colored) mites dropping onto oiled boards underneath MRCs (C). The vertical black lines at 21-Sept and 26-Sept indicate the first day we observed fighting between workers of different color morphs at the entrance to any colony (dotted line) and the first day we observed intense robbing at any colony (dashed line). Phoretic mite measurements of each MDC ended when it died.

Fig 2 shows that in the MDCs, the phoretic mite levels rose strongly in early August, peaked between mid-August and early September, and then dropped precipitously in late September, which was when the bee populations of these three colonies fell and the colonies were robbed. This figure also shows that the phoretic mite levels and the mite drop counts in the MRCs began to rise shortly after they were placed in the experimental array in mid August, and that both measures of mite abundance in the MRCs *rose* steeply between mid and late September, just when the phoretic mite levels in the MDCs *fell* steeply. The phoretic mite levels (and the mite drop counts) in the MRCs then fell to intermediate levels (10–20 mites/300 bees) in mid October and remained at these levels until they increased markedly (MRC 1E) or just slightly (all the other MRCs) in November.

For the phoretic mite levels in the MRCs, we conducted a 3 x 10 (Distance x Time) multi-way repeated measures ANOVA with a Greenhouse-Geisser correction, and found that there were no significant main effects of Time (F(1,1) = 8.57, p = 0.21) or Distance (F(1,1) = 1.27, p = 0.46), and no interaction effect (F(1,1) = 0.844, p = 0.53) (S1 Table). For the counts of adult mites dropping from each MRC, we conducted a 3 x 21 (Distance x Time) multi-way repeated measures ANOVA with a Greenhouse-Geisser correction, and found that there were no significant main effects of Time (F(1,1) = 2.20, p = 0.38) or Distance (F(1,1) = 1.15, p = 0.48), and no interaction effect (F(1,1) = 2.00, p = 0.39) (S2 Table).

Due to our small sample sizes, we also ran non-parametric analyses and investigated possible time and distance effects separately. A Friedman test found significant effects of time on the phoretic mite levels in the MRCs (*X*^*2*^(9) = 36.2, p < 0.001) and on the mite drop counts below the MRCs (*X*^*2*^(20) = 75.6, p < 0.001). Post-hoc Wilcoxon Signed-Rank tests revealed that phoretic mite levels increased over time, with significant increases from 20–30 Aug (Z = -2.03, p = 0.04) and 19–29 Sept (Z = -2.21, p = 0.03), and that there was a significant increase from the first to the last sampling timepoint (Z = -2.20, p = 0.03). Mite drop counts increased markedly between the beginning of September and October, and continued to increase through 8 Oct, with a marked drop off by 13 Oct (Z = -2.20, p = 0.03). The mite drop counts returned to baseline following this peak, with no significant difference in mite drop counts between the first and last timepoint (Z = -0.11, p = 0.92).

Based on the results of our one-way ANOVAs, we conclude that the MRCs did not differ significantly in relation to distance from the MDCs in either their peak phoretic mite levels (F(2,1) = 0.85, p = 0.51) or their peak adult mite drop counts (F(2,1) = 0.71, p = 0.56). The final phoretic mite levels of the MRCs also did not differ in relation to distance from the MDCs (F(2,1) = 1.9, p = 0.29). Non-parametric Kruskal-Wallis tests also revealed no significant differences among colonies at different distances from the MDCs, for peak phoretic mite level (*X*^2^(2) = 2.75, p = 0.28), final phoretic mite level (*X*^2^(2) = 3.71, p = 0.16), and peak mite drop count (*X*^2^(2) = 2.0, p = 0.37).

### Fighting and robbing dynamics

**Fig 3:**
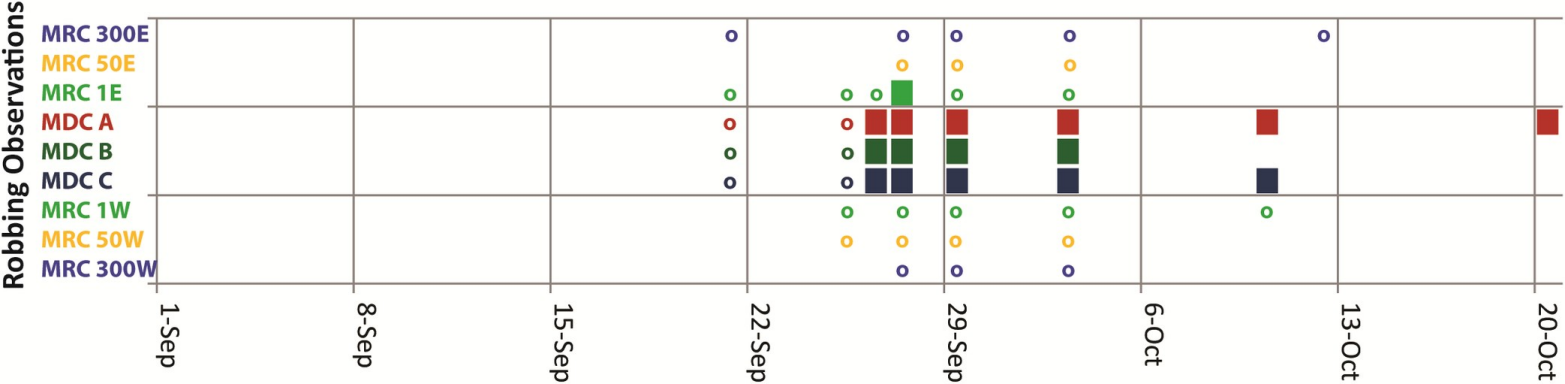
Records of fighting and robbing at MRCs and MDCs from 1 Sept to 20 Oct. An empty circle indicates when any case of worker-worker fighting at the hive entrance was seen. A filled square indicates when intense robbing of a colony was seen.

Fig 3 shows that on 21 September, which was when we first saw fighting between yellow bees and dark bees, we saw this at the hive entrances of all three MDCs, but only two of the six MRCs. It also shows that we observed intense robbing of all three MDCs by dark bees from 26 September through 4 October, which coincides with when the phoretic mite levels in the MRCs rose sharply. Indeed, the six MRCs showed marked increases in their phoretic mite levels between 10 Sept (mean of 9.0 mites per 300 bees, SD = 6.42) and 29 Sept (mean of 23.5 mites per 300 bees, SD = 11.00). A paired-samples t-test showed that phoretic mite levels increased significantly between 10 and 29 Sept (t(5) = 3.126, p = 0.026). What is most important is that four of the MRCs (1E, 50E, 300E, and 50W) had striking increases in their phoretic mite levels between 19 Sept and 29 Sept, which is when we first observed intense robbing of the MDCs by dark bees, which almost certainly came from the MRCs.

### Dynamics in worker drift from MDCs to MRCs

**Fig 4:**
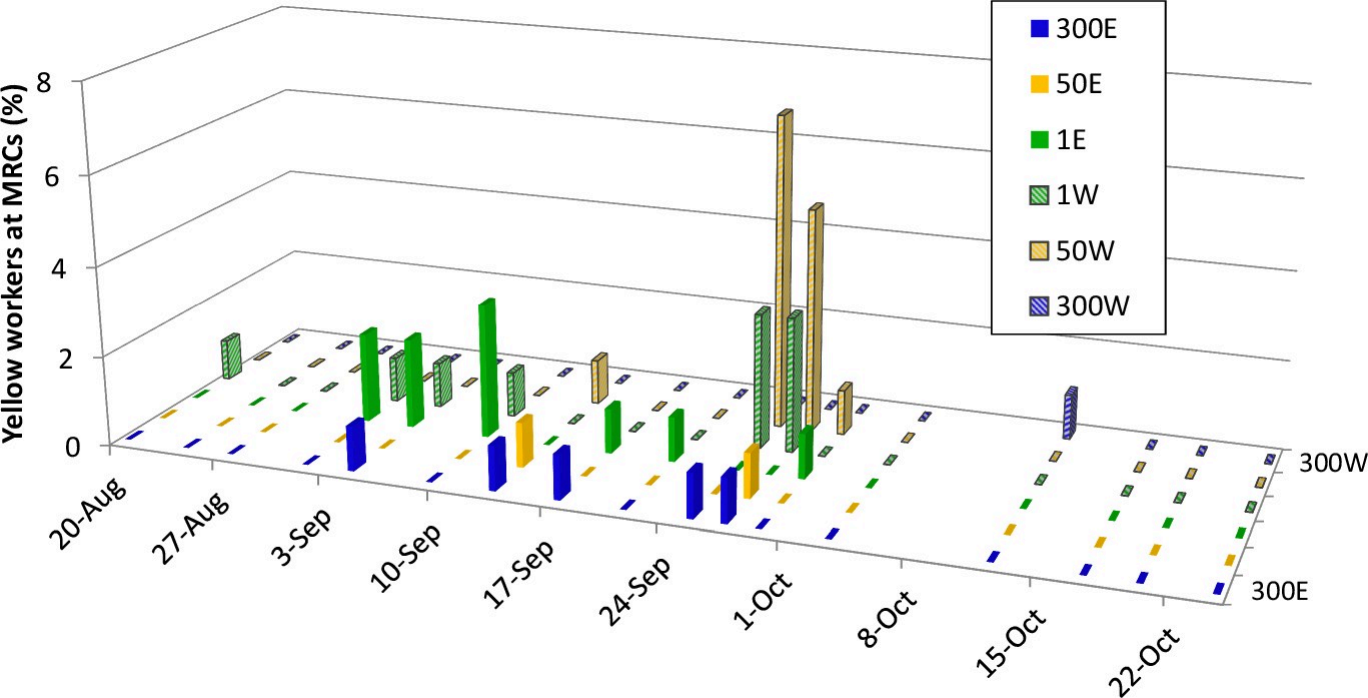
Foreign workers at MRCs. Percent of workers at the hive entrance of each MRC that were yellow (from the MDCs). Flat colored rectangles denote instances when no yellow bees were seen.

Fig 4 shows counts of bright yellow bees entering or leaving the entrances of the six MRCs, over the period of mid August to late October. In late August and early September, yellow bees were seen only occasionally entering the MRCs (mostly 1W and 1E). But on 25–27 September, when the MDCs were collapsing and being robbed by the MRCs, yellow bees were seen entering nearly all the MRCs, and especially 1W and 50W. In October, by which time the populations of all three MDCs had dwindled, there were almost no sightings of yellow workers entering the MRCs.

### Drone drift dynamics

**Fig 5:**
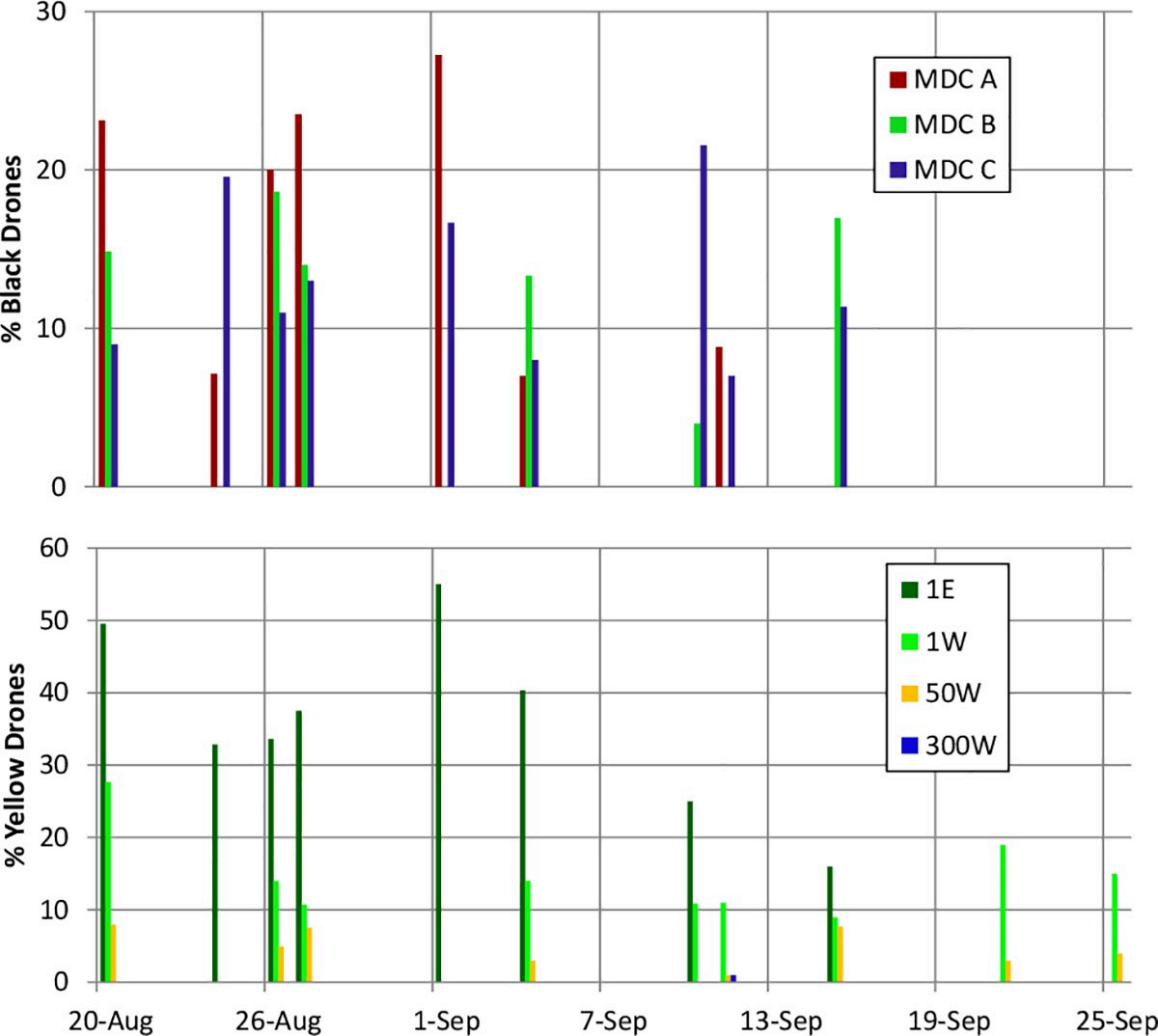
Drone drift into MDCs and MRCs over course of experiment. Data collection ended when all drones were evicted from the study colonies in late September. No yellow drones (from the MDCs) were recorded entering MRCs 300E or 50E.

Soon after we brought the MDCs and MRCs together at the study site, we began recording high levels of bi-directional drone drift between the three MDCs and the two MRCs spaced 1m from them (Fig 5). Pooling observations from nine days between 20 Aug to 15 Sept, we found that 11.7 ± 8.1% of the drones seen flying into or out of the three MDCs were black (from the MRCs). Likewise, for the same time period, 21.5 ± 16.9% of the drones flying into or out of the two MRCs located 1 m from the MDCs were yellow (from the MDCs). Meanwhile, only 0.9 ± 2.3% of the drones observed at the MRCs at 50m and 300m were yellow. Even though yellow drones (from the MDCs) were spotted flying to and from MRCs at all three distances, over the course of the study fully 92.3% of the drifted yellow drones that we observed were seen flying to and from the two MRCs located 1m from the MDCs. Before placing the MRCs in the experimental array, we had confirmed that the MDCs contained only bright yellow (Cordovan) drones, and that the MRCs contained only dark black drones.

### Dead mites and dead bees outside of hives

To document what happened as the MDCs collapsed, we placed a clean, new inverted hive cover under the entrance to each MDC on 23 August, which was 6 days after all nine colonies were assembled in the array. This action was prompted by signs of collapse in MDC A: dozens of dead adult and pupal bees, many of them with deformed wings, piling up in front of MDC A's hive. We then analyzed the contents of each cover each evening until 26 August, and determined that by the end of the third day of data collection, MDC A had disgorged 111 yellow drones, 66 yellow workers, 121 drone pupae, 11 worker pupae, and 1241 mites, of which 799 showed a mature coloration. It was not feasible to continue daily data collection at the same level of detail, but comparable numbers of dead bees and mites were found in the hive cover in front of MDC A in the following week. These numbers decreased over the following weeks as MDC A weakened. Meanwhile, over the same time period (23 and 26 August), the combined counts of disgorged bees for MDC B and MDC C were only 33 yellow drones, 22 yellow workers, 11 drone pupae, and 1 worker pupa. As the phoretic mite levels in MDC B and MDC C surged and then fell in the following weeks (Fig 2), we also noted high levels of dead bee and dead mite outflow like what we had seen with MDC A. Thus, we found dead yellow bees (workers and drones) with shriveled or malformed wings in front of all three MDCs as they collapsed, consistent with high levels of infection with mite-transmitted bee viruses.

The number of dead bees piling up outside the entrances of the MDC's hives suddenly increased a second time, during the period of intense robbing in late September (Fig 3), by which time all three MDCs had become weak and the fall honey flow from goldenrod (*Solidago* spp.) had begun to weaken. Between 25–27 September, the inverted hive cover in front of MDC A accumulated 500 (±5) dead workers, 90% of them dark (foreign bees); that of MDC B accumulated 1340 (±5) dead workers, 88% dark; and that of MDC C accumulated 215 (±5) workers, 72% of them dark. For comparison, between 25–27 September, the inverted hive covers in front of MRCs 1E and 1W accumulated 175 (±5) and 185 (±5) dead workers, only 7% and 6% of them yellow (foreign bees), respectively.

### Tracking robbers home by sugar dusting

On 27 September, a day with intense robbing (Fig 3) of all three MDCs, we dusted the bees at the entrances of the MDCs' hives with powdered sugar, and then we watched at the entrance of each MRC's hive for dusted bees entering there. We evaluated 200 returning workers per MRC. We counted five dusted bees entering MRC 50E, four entering both MRCs 300E and 300W, and two each entering MRCs 50W, 1W, and 1E.

### Deaths of colonies

The three MDCs died in early autumn: by 10 Oct (MDC C), by 19 Oct (MDC B), and by 2 Nov (MDC A). After the deaths of the MDCs, we checked the MRCs bimonthly to monitor their winter mortality. MRC 1E was dead by 20 Dec 2017. MRCs 1W, 300W, and 300E were dead by 20 Feb 2018. Both MRCs 50W and 50E survived to the spring of 2018. No colonies showed signs of impending queen failure before winter, and examination of all dead colonies in April revealed no detectable signs of starvation or moisture damage, which suggests that mites and mite-associated viruses may have killed the four MRCs that perished.

## Discussion

The goal of this study was to investigate the processes that underlie the "mite bomb" phenomenon: when some colonies collapse from high levels of Varroa mites, other colonies nearby often experience surges in their levels of mites. The findings reported here—(1) the dynamics of the Varroa mite counts in the MDCs and the MRCs, (2) the robbing of the MDCs by workers from the MRCs, and (3) the drifting of workers and drones from the MDCs to the MRCs—document the pattern of events associated with a carefully observed instance of the "mite bomb" phenomenon. These findings also shed light on the behaviors of the bees that produce this phenomenon.

Regarding the pattern of events, we report what happened when three colonies with high mite counts (the MDCs) and six colonies with low mite counts (the MRCs) were brought together in the middle of August. We saw that a few weeks later, starting around mid September, the phoretic mite counts in the MDCs plummeted while at the same time these mite counts in the MRCs rose steeply (Fig 2). Between 10 September and 29 September, the phoretic mite counts of the MDCs decreased by an average of 37 mites per 300 bees, while those of the MRCs increased by an average of 14.5 mites per 300 bees.

What processes produced this pattern of surging mite loads in the MRCs? Our data suggest strongly that the primary process was robbing of the collapsing MDCs by workers from the MRCs (Fig 3), but that drifting of mite-infested workers and drones from the MDCs to the MRCs was also important, especially for the MRCs closest to the MDCs (Figs 4 and 5). That robbing played a major role in producing the surges in the mite loads of the MRCs is indicated by three important things that happened *simultaneously* in late September: (1) the MDCs lost strength to the point where they were intensely robbed by workers from the MRCs, (2) the mite loads of the MDCs plummeted, and (3) the mite loads of the MRCs surged.

In early summer, a high percentage of a colony’s mite population is found reproducing in cells of capped brood, but in late summer, these mites become mainly phoretic as the brood production of their host colony tapers off [25]. It is possible that some of the late summer increases in mite loads that we observed in our MRCs (and that others have reported in their colonies) may be driven by this phenomenon. However, the points enumerated above support the hypothesis that robbing was a significant driver of the rapid increases in phoretic mite loads that we observed in late summer/early autumn.

Further evidence that robbing of the collapsing colonies was responsible for the movement of large numbers of mites into the MRCs comes from what we learned by sugar dusting robbers at the MDCs: workers from every MRC were engaged in robbing the MDCs. Evidently, the MRCs acquired mites regardless of distance from the MDCs. Indeed, the most dramatic post-robbing spike in phoretic mite load was seen in MRC 300E, one of the two MRCs farthest from the MDCs. Evidently, intercolony distances of 300m (or perhaps considerably more) offer colonies little protection from acquiring mites through robbing.

Our findings suggest that drifting also played a role in mite transmission from collapsing colonies to healthy ones, particularly to those adjacent to the collapsing colonies. Of the two time periods for which our post-hoc tests revealed significant increases in the phoretic mite levels of the MRCs, one occurred soon after the introduction of the MRCs to the experimental array (20–30 Aug), a time when drift was the only likely transmission mechanism. Before robbing of the MDCs became intense at the end of September, most of the drifting by workers and drones from the MDCs was into MRCs 1E and 1W (Figs 4 and 5). On 10 Sept, before we had observed either minor robbing (worker-worker aggression at hive entrances) or intense robbing of the MDCs, the MRCs 1m from the MDCs had higher phoretic mite loads than did the colonies 50m and 300m away. This pattern might have persisted and expanded had robbing not begun. That this pattern in the data vanished as soon as robbing began lends support to the conclusion that while drifting (particularly to nearby colonies) can be a mechanism for mite transmission between colonies, the effects of drifting can be rapidly overshadowed by the strong dispersal of mites via robbing.

The highest drifting of drones into the MRCs was seen the day after the MRCs were moved to the study site, and this drifting of drones was almost exclusively into the MRCs 1m from the MDCs (Fig 5). The short range of this drifting suggests that drones probably drifted due to orientation errors, and not as part of an adaptive invasion of neighboring colonies. Despite the low levels of drifting, the steady rise in the mite levels of the MRCs from when the experiment started to when the robbing began (Figs 2 and 3) suggests that drifting by mite-infested workers, mite-infested drones [26], or both, facilitated mite migration into the nearby colonies (MRCs) before the onset of robbing. We regularly observed a few drifted (yellow) workers from the MDCs in the MRCs 300m away. This indicates that to completely protect a colony from acquiring Varroa mites through worker and drone drifting, one needs intercolony spacings even greater than 300m.

We saw no indications that drifting of bees from the MDCs into the MRCs increased as the mite levels in the MDCs increased (for workers, see Fig 4; for drones, see Fig 5). Although phoretic mites typically infest younger workers, some can be found infesting older workers functioning as foragers [27], especially when phoretic mite infestations climb above 20% [28]. Some have proposed that these mites may be adapted to disperse by selectively infesting foragers, and that they may even increase the rate at which these infested foragers drift by impeding their orientation [12]. This is the concept of a “mite bomb” colony, suddenly “blasting” mite-infested, drift-prone worker bees into neighboring colonies like shrapnel from an exploding bomb. Our findings (Figs 4 and 5), however, support those of Goodwin et al. [17], who found no dramatic increase in worker drift during mite-induced deaths of colonies, and instead found relatively constant rates of worker drift out of the dying colonies. We observed only one dramatic, but extremely brief, increase in the number of yellow workers entering a few of the MRCs (Fig 4), and it occurred in late September, precisely when we also observed intense robbing of the MDCs (Fig 3). Given our study's methods, we cannot say for sure whether these yellow (MDC) workers in the MRCs’ hives got there by drifting or by robbing, but we note that this brief increase happened at the onset of intense robbing in the array, not the time of peak mite counts in the MDCs.

There are two reasons why we performed this experiment with a cluster of three MDCs instead of just one. First, the use of three MDCs increased the potential mite influx we could observe in the six MRCs. Second, the use of three MDCs increased the likelihood that at least one colony would collapse during our observations. The use of three MDCs means, however, that, some of the bees and mites leaving each MDC might have entered the two neighboring MDCs. Therefore, we cannot tie specific mite level increases in the MRCs to specific changes in any one MDC. Future studies must balance the need to observe mite-induced colony collapse and the desire to assign individual drifting or robbing bees to their natal hives.

This study produced detailed data from a small number of colonies. The labor-intensive methodology that we used revealed how various behaviors of individual honey bees produced mite transmission between colonies, but this methodology also limited the number of colonies that we could study. The patterns we observed regarding distance from the MDCs, levels of worker and drone drift, and mite levels in the MRCs, are essentially symmetrical for the east and west sectors of our experimental array, so our study has a built-in replication. Moreover, we traced robbers back to each of our six MRCs as their mite levels climbed, so we also report multiple instances of direct evidence of robbing playing a role in mite transmission between colonies. However, given our small sample sizes this case study cannot be used to draw absolute conclusions about the relationship between drift, robbing, and mite transmission without further investigation. Future research on this subject using more colonies will no doubt yield additional insights into how bee behavior drives intercolony mite transmission, and increased sample sizes may reveal subtleties about effects of intercolony distance or other factors that we could not detect with our small sample sizes. In addition, because it has been observed that bees in some locations do not readily rob weak colonies in the autumn (Randy Oliver, pers. comm.), it is entirely possible that the robbing-driven “mite bomb” phenomenon that we observed in New York state may differ from the “mite bomb” phenomenon in other places, where drifting may play a larger role, or robbing a smaller one.

Our findings confirm that colonies that are heavily infested with mites pose a serious risk of transmitting their mites to nearby colonies. Our data also reveal that the colloquial terminology for these colonies—“mite bombs”—does not accurately describe the mechanisms of intercolony mite transmission that we observed in this study. We saw no sudden “explosion” of mite-carrying bees from sick colonies to each of the healthy colonies via worker drifting. Indeed, we found that few mites passed from the heavily infested (MDC) colonies to the weakly infested (MRC) colonies through either worker drift (mostly to nearby colonies, Fig 4) or drone drift (almost exclusively to nearby colonies, Fig 5). It was only when the MDCs were weakened so much that they became irresistible robbing targets that mites passed in large numbers from the MDCs to the MRCs. We suggest, therefore, that “robber lures” is a better term than “mite bombs” to describe mite-source colonies, especially in contexts where weak colonies are readily robbed by neighboring hives. The distinction between “mite bombs” and “robber lures” is important for understanding the optimal virulence of the mites and the viruses they vector. If the sickness of a host increases the opportunities for its parasite to spread, then the parasite should evolve higher virulence [29, 30, 31]. In our study, we found that the mites spread to neighboring colonies primarily through the robbing of very sick (collapsing) colonies. It is possible that natural selection has favored, and will continue to favor, strains of mites and mite-borne viruses that severely weaken the defenses of their host colonies to make them attractive targets to robber bees.

## Supporting information

S1 Dataset Digital spreadsheet of behavioral observations and parasite data reported in this manuscript. (XLSX)

S1 Table Phoretic mite count multi-way repeated measures ANOVA results table. (XLS)

S2 Table 48-hr Mite drop multi-way repeated measures ANOVA results table. (XLS)

S1 Video Intense robbing at the hive entrance of a mite-weakened MDC. Recording made on 27 September of dark-colored MRC robber bees fighting with light-colored MDC guard bees at the entrance to one of the MDC colonies. It shows the chaotic nature of an intense bout of robbing. This recording also shows that the two color-morphs of bees are easily distinguished.
(MP4)
